# Occupational Exposure to Vapor-Gas, Dust, and Fumes in a Cohort of Rural Adults in Iowa Compared with a Cohort of Urban Adults

**DOI:** 10.15585/mmwr.ss6621a1

**Published:** 2017-11-03

**Authors:** Brent C. Doney, Paul K. Henneberger, Michael J. Humann, Xiaoming Liang, Kevin M. Kelly, Jean M. Cox-Ganser

**Affiliations:** 1 Respiratory Health Division, National Institute for Occupational Safety and Health, CDC, Morgantown, West Virginia; 2 The University of Iowa, Department of Occupational and Environmental Health, Iowa City, Iowa

## Abstract

**Problem/Condition:**

Many rural residents work in the field of agriculture; however, employment in nonagricultural jobs also is common. Because previous studies in rural communities often have focused on agricultural workers, much less is known about the occupational exposures in other types of jobs in rural settings. Characterizing airborne occupational exposures that can contribute to respiratory diseases is important so that differences between rural and urban working populations can be assessed.

**Reporting Period:**

1994–2011.

**Description of System:**

This investigation used data from the baseline questionnaire completed by adult rural residents participating in the Keokuk County Rural Health Study (KCRHS). The distribution of jobs and occupational exposures to vapor-gas, dust, and fumes (VGDF) among all participants was analyzed and stratified by farming status (current, former, and never) then compared with a cohort of urban workers from the Multi-Ethnic Study of Atherosclerosis (MESA). Occupational exposure in the last job was assessed with a job-exposure matrix (JEM) developed for chronic obstructive pulmonary disease (COPD). The COPD JEM assesses VGDF exposure at levels of none or low, medium, and high.

**Results:**

The 1,699 KCRHS (rural) participants were more likely to have medium or high occupational VGDF exposure (43.2%) at their last job than their urban MESA counterparts (15.0% of 3,667 participants). One fifth (20.8%) of the rural participants currently farmed, 43.1% were former farmers, and approximately one third (36.1%) had never farmed. These three farming groups differed in VGDF exposure at the last job, with the prevalence of medium or high exposure at 80.2% for current farmers, 38.7% for former farmers, and 27.4% for never farmers, and all three percentages were higher than the 15.0% medium or high level of VGDF exposure for urban workers.

**Interpretation:**

Rural workers, including those who had never farmed, were more likely to experience occupational VGDF exposure than urban workers.

**Public Health Action:**

The occupational exposures of rural adults assessed using the COPD JEM will be used to investigate their potential association with obstructive respiratory health problems (e.g., airflow limitation and chronic bronchitis). This assessment might highlight occupations in need of preventive interventions.

## Introduction

Chronic obstructive pulmonary disease (COPD) is an inflammatory lung disease that impedes airflow and is a major cause of morbidity and mortality worldwide (*1*–*3*). The American Thoracic Society estimated that 15% of COPD cases in the general population can be attributed to occupational exposures (*4*,*5*). Specifically, occupational exposures to vapor-gas, dust, and fumes (VGDF) have been associated with COPD (*4*,*6*). Investigating differences between rural and urban workers in respiratory disease outcomes (including COPD) first requires characterization of occupational VGDF exposures overall. Although rural communities have many agriculture-related jobs, which have received the focus of previous studies, employment in nonagricultural jobs also is common; therefore, VGDF exposures both in agricultural and nonagricultural jobs in rural communities should be assessed.

Occupational exposure to VGDF can be assessed by various methods. One method is a job-exposure matrix (JEM), which eliminates reporting bias that might occur with self-reports of occupational exposure (*7*). An expert-based JEM developed by CDC specifically for COPD risk (COPD JEM) provides a method to assess overall VGDF exposure and subcomponents of VGDF, including vapor-gas, total dust, mineral dust, organic dust, and fumes. The distribution of occupational groups and occupational exposures to VGDF among workers in a rural county was analyzed and contrasted with the occupational groups and occupational exposures from a previous study of workers in urban communities (*8*). This report provides insight into differences in occupational exposures in a rural and an urban population. Data can be used by epidemiologists and other researchers to identify areas for additional study and by occupational health professionals, industrial hygienists, and policy makers to identify opportunities for interventions.

## Methods

Data in this report were obtained from the baseline questionnaire completed by adults aged ≥18 years in the Keokuk County Rural Health Study (KCRHS), a community-based prospective cohort study of residents in a rural Iowa county, with a 2010 population of 10,500. Data were collected in three rounds of testing during 1994–2011 (*9*). Round 1 was administered during 1994–1998, round 2 during 1999–2004, and round 3 during 2006–2011. The questionnaire asked participants about all current and previous occupations. The free-text occupational information was used by CDC expert coders to assign a 2002 U.S. Census occupational code (COC) to each participant’s last reported job. Occupational exposure was assigned to each occupational code using COPD JEM.

COPD JEM was constructed by an industrial hygienist who assessed the likelihood and severity of exposure to total VGDF and assigned a total VGDF exposure score of none or low (score of 1), medium (score of 2), or high (score of 3) to each COC (*8*). The hygienist similarly assigned separate exposure scores for the subcomponents of VGDF (i.e., vapor-gas, total dust, mineral dust, organic dust, and fumes). These preliminary exposure scores were reviewed by two certified industrial hygienists, and then all three experts held meetings and assigned a final consensus exposure score.

Cross-sectional results from the Multi-Ethnic Study of Atherosclerosis (MESA) cohort study of urban workers (data collected during 2000–2002) were used for comparison (*8*). MESA is a population-based sample of adults aged 45–84 years from six primarily large, urban communities (Baltimore, Maryland; Chicago, Illinois; Forsyth County, North Carolina; Los Angeles, California; New York City, New York; and St. Paul, Minnesota). With the exception of Forsyth County in North Carolina, the 2010 populations for these six areas were all >1 million: 805,000 for Baltimore City and Baltimore County, Maryland, and 2.8 million for the greater Baltimore metropolitan area; 5.2 million for Chicago, Illinois; 351,000 for Forsyth County, North Carolina; 9.8 million for Los Angeles County, California; 8.2 million for New York City, New York; and 509,000 for St. Paul, Minnesota, with 1.2 million for the twin cities of St. Paul and Minneapolis. As with KCRHS, CDC expert coders assigned a COC to each MESA study participant’s last reported job and applied the COPD JEM (*8*). The occupational groups are major groups of occupations (used in previous MESA occupational studies) and are the same for both studies (management/professional, COC 0010–3540; service, COC 3600–4650; sales/office, COC 4700–5930; and blue collar, COC 6000–9750). Therefore, the characterization of occupational exposure for rural and urban workers was similar. The 95% confidence intervals (CIs) for percentages were calculated using a simple asymptotic method (*10*). 

The KCRHS study protocol was approved by the Institutional Review Board of the University of Iowa, and the project was approved by the CDC Institutional Review Board. Written informed consent was obtained from each KCRHS participant.

## Results

The 1,699 adult rural residents who participated in KCRHS and provided complete baseline data had a mean age of 51.2 years (standard deviation [SD]: 17.0, range: 18–92 years) at enrollment, and the mean birth year was approximately 1946. Among the participants, 961 (56.6%) were women, 15.2% were current smokers, and 23.7% were former smokers (Table 1). In the MESA study of urban adults (n = 3,667), the mean age was 61.1 years (SD: 9.8, range: 45–84 years), mean birth year was approximately 1940, a total of 1,789 (48.8%) were women, 9.8% were current smokers, and 45.2% were former smokers. 

**TABLE 1:** Characteristics of participants in the Multi-Ethnic Study of Atherosclerosis (2000–2002) and the Keokuk County Rural Health Study (1994–2011), by farming status of rural participants

Characteristic	MESA (urban) (n = 3,667)	KCRHS (rural)			
		Total (n = 1,699)	Current farmer (n = 353)	Former farmer (n = 732)	Never farmer (n = 614)
	% (95% CI)	% (95% CI)	% (95% CI)	% (95% CI)	% (95% CI)
**Mean age, yrs (95% CI)**	**61.1 (60.8–61.4)**	**51.2 (50.4–52.0)**	**49.1 (47.5–50.8)**	**53.2 (52.0–54.4)**	**50.0 (48.6–51.4)**
**Sex**					
Female	48.8 (47.2–50.4)	56.6 (54.2–58.9)	27.5 (23.1–32.4)	54.8 (51.2–58.4)	75.4 (71.9–78.7)
Male	51.2 (49.6–52.8)	43.4 (41.1–45.8)	72.5 (67.6–76.9)	45.2 (41.7–48.8)	24.6 (21.4–28.2)
**Smoking status**					
Never	45.1 (43.4–46.7)	61.1 (58.8–63.4)	65.1 (60.1–69.9)	57.5 (53.9–61.0)	63.0 (59.1–66.8)
Former	45.2 (43.5–46.8)	23.7 (21.7–25.7)	21.0 (17.0–25.5)	27.6 (24.5–31.0)	20.5 (17.5–23.9)
Current	9.8 (8.8–10.8)	15.2 (13.6–17.0)	13.9 (10.7–17.9)	14.9 (12.5–17.7)	16.5 (13.7–19.6)
**Occupational group for last job**					
Management/professional	44.3 (42.7–45.9)	32.7 (30.5–35.0)	55.2 (50.0–60.3)	28.3 (25.1–31.7)	25.1 (21.8–28.7)
Blue-collar	19.3 (18.1–20.7)	25.8 (23.8–28.0)	30.6 (26.0–35.6)	30.5 (27.2–33.9)	17.6 (14.8–20.8)
Sales/office	20.8 (19.5–22.2)	22.4 (20.5–24.5)	7.4 (5.1–10.6)	23.9 (21.0–27.1)	29.3 (25.9–33.0)
Service	15.5 (14.4–16.8)	14.9 (13.3–16.7)	5.4 (3.5–8.3)	12.0 (9.9–14.6)	23.8 (20.6–27.3)
Other	0	4.1 (3.3–5.2)	1.4 (0.6–3.3)	5.3 (3.9–7.2)	4.2 (2.9–6.1)
**Total VGDF exposure for last job**					
None or low	85.0 (83.8–86.1)	56.8 (54.4–59.1)	19.8 (16.0–24.3)	61.3 (57.8–64.8)	72.6 (69.0–76.0)
Medium	9.8 (8.9–10.8)	17.5 (15.8–19.4	9.9 (7.2–13.5)	20.1 (17.3–23.1)	18.9 (16.0–22.2)
High	5.3 (4.6–6.0)	25.7 (23.7–27.8)	70.3 (65.3–74.8)	18.6 (15.9–21.6)	8.5 (6.5–10.9)

**Abbreviations:** CI = confidence interval; KCRHS = Keokuk County Rural Health Study; MESA = Multi-Ethnic Study of Atherosclerosis; VGDF = vapor-gas, dust, and fumes.

Compared with urban (MESA) participants, rural (KCRHS) participants were younger by an average of approximately 10 years, somewhat more likely to be women, more likely be current smokers, and less likely to be former smokers. For the last reported job, rural participants (compared with urban participants) had a lower proportion of management/professional jobs (32.7% versus 44.3%) and a higher proportion of blue-collar jobs (25.8% versus 19.3%) (Table 1). The proportion of sales/office jobs and service jobs was similar in the two populations. Regarding VGDF exposure, rural participants were more likely to have medium or high occupational total VGDF exposure than the urban participants, with percentages of 17.5% versus 9.8% for medium exposure and 25.7% versus 5.3% for high exposure, respectively, or 43.2% versus 15.0% for medium and high exposure combined. Therefore, the rural-urban ratio of total VGDF exposure was approximately two for medium exposure and five for high exposure.

Rural participants included 353 (20.8%) who were current farmers, 732 (43.1%) who had previously farmed, and 614 (36.1%) with no farming experience. The farming groups varied by age and smoking habits; former farmers were older and more likely to have ever smoked cigarettes (Table 1). The farming groups also differed by sex; the percentage of men ranged from 72.5% among current farmers to 45.2% among former farmers to 24.6% among never farmers. VGDF exposure varied by farming status. High VGDF exposure in the last job decreased from current farmers (70.3%) to former farmers (18.6%) to never farmers (8.5%), and no or low exposure increased from current farmers (19.8%) to former farmers (61.3%) to never farmers (72.6%) (Table 1). Similar trends by farming status also were observed for the COPD JEM exposures for VGDF subcomponents (data not shown). Similar to what was observed when comparing all rural participants to urban participants, those in each farming status group were more likely to have experienced total VGDF exposure in their last job than the urban workers. The prevalence of medium or high exposure combined was 80.2% among current farmers, 38.7% among former farmers, and 27.4% among never farmers, versus 15.0% for the urban cohort.

The frequency of medium or high exposure combined in the rural cohort was higher for vapor-gas (36.5%) than total dust (30.8%). Among the two dust subcomponents, organic dust exposure (25.3%) was more common than mineral dust exposure (16.7%) (Table 2). For total VGDF, rural participants were more likely to experience exposure than urban participants, a finding that was evident for each of the VGDF subcomponents except for fumes. In addition, these differences were a result of the greater percentage of KCRHS participants in the high exposure categories.

**TABLE 2:** Percentage of participants in the Multi-Ethnic Study of Atherosclerosis (2000–2002) and the Keokuk County Rural Health Study (1994–2011) who were exposed to subcomponents of vapor-gas, dust, and fumes, by likelihood and severity of exposure

Likelihood and severity of exposure*	MESA (urban) (n = 3,667)	KCRHS (rural) (n = 1,699)
	% (95% CI)	% (95% CI)
**Vapor-gas**		
None or low	82.5 (81.2–83.7)	63.5 (61.2–65.8)
Medium	13.3 (12.2–14.4)	14.1 (12.6–15.9)
High	4.3 (3.6–5.0)	22.4 (20.5–24.4)
**Total dust**		
None or low	88.0 (86.9–89.0)	69.2 (66.9–71.3)
Medium	9.2 (8.3–10.1)	9.7 (8.4–11.1)
High	2.8 (2.3–3.4)	21.2 (19.3–23.2)
** Mineral dust**		
None or low	94.7 (94.9–95.4)	83.3 (81.4–85.0)
Medium	4.1 (3.5–4.8)	3.7 (2.9–4.7)
High	1.2 (0.9–1.6)	13.1 (11.6–14.8)
** Organic dust**		
None or low	92.4 (91.5–93.2)	74.8 (72.6–76.8)
Medium	5.9 (5.2–6.7)	5.9 (4.9–7.1)
High	1.7 (1.3–2.2)	19.4 (17.6–21.3)
**Fumes**		
None or low	95.4 (94.7– 96.1)	94.9 (93.8–95.9)
Medium	3.1 (2.6–3.7)	2.7 (2.0–3.6)
High	1.5 (1.1–1.9)	2.4 (1.7–3.2)

**Abbreviations:** CI = confidence interval; KCRHS = Keokuk County Rural Health Study; MESA = Multi-Ethnic Study of Atherosclerosis.

*The chronic obstructive pulmonary disease job exposure matrix (COPD JEM) was used to assess likelihood and severity of exposure.

## Discussion

Unlike other studies that focus exclusively on agricultural jobs in rural settings, this report examined occupational exposures in all types of jobs in one rural community. The findings indicated that workers in a rural Iowa county were more likely to have occupational VGDF exposure than a cohort of urban workers from various cities. Although certain studies have compared rates of obstructive respiratory disease in rural and urban cohorts (*11*–*15*), this study assessed occupational exposures that might help explain some differences in disease frequency. In future analyses, CDC researchers will use KCRHS data to investigate whether obstructive respiratory outcomes, such as airflow limitation and chronic bronchitis, are associated with the occupational exposures identified by COPD JEM.

Although the direct measurement of exposure usually is considered the ideal approach for assessing occupational exposures, direct measurement seldom occurs (*16*). JEMs are a useful option when other exposure assessment strategies, such as air monitoring, are not possible, which is often the case in population-based studies. Unlike direct measurement of exposures, JEMs cannot account for exposure variability within jobs (*16*). JEMs have been used extensively for cancer outcomes and also have been used to study nonmalignant respiratory diseases such as COPD (*17*) and asthma (*18*,*19*). Although obtaining self-reports of occupational exposures is easier than directly measuring exposures or developing JEMs, it might bias effect estimates (e.g., when those with the health outcome of interest overreport exposure and those without the health outcome underreport exposure). In a study of asthma, although self-reported exposure was more common in areas where asthma was more prevalent, the difference was not observed for JEM-assessed exposure (*7*). A review of occupational COPD and JEMs found that self-reports of occupational exposure might lead to overestimations of the risk for occupational COPD (*17*).

Differences in age between the rural (KCRHS) and urban (MESA) populations were a result of the respective study designs. KCRHS was intended to include a full age range of adults, and MESA was designed to include older adults who were aged ≥45 years at recruitment. The younger average age of rural participants likely contributed to the group’s lower percentage of former smokers. For example, national data from the 2004 Behavioral Risk Factor Surveillance System survey found that the frequency of former smokers increased with age (*20*). The higher percentage of current smokers among rural participants is consistent with national trends of higher smoking rates in younger adults (*21*,*22*) and in rural versus urban communities (*12*,*15*,*21*). 

After the original analysis described in the methods was conducted, a post hoc sensitivity analysis was conducted to examine whether age affected certain differences between rural and urban participants. The rural sample was limited to the 1,049 participants who were aged ≥45 years, comparable with the age range of the urban cohort. These older rural KCRHS participants were somewhat more likely to be former smokers and less likely to be current or never smokers than the entire cohort, although the differences with MESA persisted. In addition, the 1,049 older members of the rural cohort had nearly the same frequency distributions by sex and by occupational group and exposures as all 1,699 participants. Therefore, the differences in occupations and JEM-assessed exposures with MESA were the same for the older KCRHS participants as for the entire cohort, suggesting that the different age groups did not affect these results.

In this report, the proportion of management/professional and blue-collar jobs differed between the rural and urban cohorts, with a higher proportion of management/professional and a lower proportion of blue-collar workers among the urban residents. However, the subset of current farmers in the rural cohort had a higher percentage in the management/professional job group than the urban cohort, with 55.2% versus 44.3%, respectively (Table 1). On the basis of how current farmers described their work, many were assigned to the job categories of 1) farm, ranch, and other agricultural managers and 2) farmers and ranchers, which were included in the management/professional occupational group. Others who conducted agricultural work were coded in the job of miscellaneous agricultural worker that was included in the blue-collar group. The blue-collar group (COC 6000–9750) also included nonfarm agricultural jobs such as graders and sorters of agricultural products, as well as construction workers, skilled trade workers, and laborers.

Although current and former farmers accounted for 63.9% of the rural cohort, they represented 88.1% of rural participants with high total VGDF exposure in the last job. Therefore, any findings of an association between respiratory health and high VGDF exposure might be primarily a result of farming exposures, and determining whether an exposure-response relation is evident both for those with and those without farming experience is important.

## Limitations

The findings in this report are subject to at least four limitations. First, although the rural cohort is relatively large, it includes only one rural county and is not representative of the entire U.S. rural population. Second, five of the six metropolitan areas in the urban cohort had populations >1 million, several were very large, and the areas did not include smaller cities, with the exception of Forsyth County, North Carolina. In addition, the urban cohort did not include participants from cities in Iowa, the largest of which is Des Moines in Polk County, with a 2010 population of 430,600. The rural-urban comparison of occupational VGDF exposure in this report might have differed if the urban cohort were dominated by smaller cities and/or cities in Iowa. Third, small numbers for occupational exposure subcomponents might limit subsequent analyses of health outcomes for the rural cohort, most notably for fumes. Finally, air measurements and self-reports were not used to characterize occupational exposures across the full range of jobs in this rural setting. However, a recently developed JEM was applied, and the similarity of exposure-assessment methods with the urban cohort facilitated the rural-urban comparison.

## Conclusion

Rural residents were more likely to have high occupational exposures to total VGDF than urban residents. This was true even for rural residents who did not have farm experience. The distribution of occupational exposures as assessed by COPD JEM in the cohort of rural adults makes it possible to subsequently explore exposure-response relations between VGDF and obstructive respiratory outcomes, such as airflow limitation and chronic bronchitis.
